# Anxiety and Stress During the COVID-19 Lockdown Among the General Population in Perambalur: A Cross-Sectional Study

**DOI:** 10.7759/cureus.34411

**Published:** 2023-01-30

**Authors:** Tamilarasan M, Karthikeyan Kulothungan, Nawin Vignesh, Neethu George, Rock B Dharmaraj

**Affiliations:** 1 Community Medicine, Dhanalakshmi Srinivasan Medical College and Hospital, Perambalur, IND

**Keywords:** stress, anxiety, coronavirus disease 2019, covid-19, coronavirus anxiety scale, generalized anxiety disorder scale, perceived stress scale, corona anxiety stress scale, mental health

## Abstract

Background

The coronavirus disease 2019 (COVID-19) pandemic and lockdown had a significant impact on mental health during the last two years. However, the majority of studies do not concentrate on the risk and protective factors that influence the relationship between COVID-19 and subjective well-being. Therefore, the present study aims to identify such stressful experiences and the influence of COVID-19 and various stressors.

Methodology

We have conducted this community-based, cross-sectional, analytical study in the Perambalur district of Tamil Nadu for four months. After obtaining approval from the Institutional Ethics Committee, we gathered data for the study. Two field practice areas were involved in data collection. A convenient sampling procedure was used to select 291 households for the study. The lead investigator interviewed one individual from each household, preferably the head of the family. A semi-structured questionnaire was used to collect the pertinent information. The Coronavirus Anxiety Scale (CAS), Perceived Stress Scale (PSS), and Generalized Anxiety Disorder (GAD) scale were used to assess anxiety and stress. All collected data were entered into Microsoft Excel (Microsoft Corporation, Redmond, WA), and SPSS software version 21 (IBM Corp., Armonk, NY) was used to analyze the results.

Results

Among the participants, 34% had a history of COVID-19 infection, and 58.4% of the families had at least one chronic comorbidity among the family members. The CAS score was significantly associated with the residence (p = 0.049), marital status (p = 0.001), and previous history of COVID-19 (p = 0.016) of the study participants. The study found that gender was the only factor associated with both the PSS score (p = 0.022) and the GAD scale score (p = 0.010) of the study participants.

Conclusions

Even though doctors can treat many mental health illnesses for a comparatively minimal cost, there is still a wide disparity between those who require care and those who have access to it. Governmental programs and regulations that conduct routine surveys to identify anxiety and stress can lead to successful preventative strategies.

## Introduction

Emotional, psychological, and social well-being are all parts of human mental health [1]. It impacts how we feel, think, and behave. More than just being free of mental illness, we can see mental health as a state that allows one to flourish and fully enjoy life [2].

In recent years, there has been a rising understanding of the critical role that mental health plays in accomplishing global development goals, as demonstrated by the inclusion of mental health in the Sustainable Development Goals [3]. Curable physical conditions can cause people with severe mental diseases to die up to two decades early [4].

According to the WHO, 20% of children and adolescents globally have mental health problems [5]. According to the Global Burden of Disease study report from 2017, slightly more than one in 10 people (10.7%) live with a mental health disorder [6].

Everyone goes through tough situations in life. The capacity to deal with unpleasant situations varies widely from person to person and influences whether people enjoy their life [7].

A person's mental health may be affected if the demands placed on them are greater than their capacity for coping [8]. For instance, someone may have poor mental health if they are working long hours, providing care for a relative, or going through financial difficulties.

The lockdown because of the coronavirus disease 2019 (COVID-19) pandemic had a significant impact on mental health over the last two years. An unprecedented worldwide public health emergency is being created by the psychological consequences of the COVID-19 pandemic [9]. The extended lockdown and necessary safety measures increased uncertainty and changed several facets of day-to-day living.

Some of the earlier studies conducted on the prevalence of mental health issues revealed that symptoms of stress, depression, and post-traumatic stress were fairly common and high [10-12]. However, most research studies do not concentrate on the risk and protective factors that influence the link between stressful events and subjective well-being. Therefore, the present study aims to identify such stressful experiences and the influence of COVID-19 and various stressors.

## Materials and methods

Study design

The present study is a community-based, cross-sectional, analytical study.

Study population, place, and duration

We collected the data through a house-to-house survey using the interview method during the period of July 2021 to October 2021 in the Perambalur district of Tamil Nadu.

Ethical clearance and informed consent

Before conducting the study, we obtained an ethical clearance certificate from the Institutional Ethics Committee of Dhanalakshmi Srinivasan Medical College and Hospital (approval number: IECHS/IRCHS/No. 122). We explained the objectives of the study to participants before they gave their consent to participate.

Inclusion and exclusion criteria

Two field practice areas were involved in data collection. One field practice area was urban, and the other was rural. We interviewed the head of the family or their spouse from each household. If both persons were not present during the time of the survey or if the house was locked, we visited the same house on another day to collect data. Members who were not present in the house for more than two visits were excluded from the study.

Sample size

According to a previous study conducted in India by Srivastava et al. [13], the prevalence of COVID-19-related psychological disorders was 53.3%. The current study's sample size was calculated using a single proportion sample size determination formula (Z1-α/2)2*p*q/d2), with a prevalence of psychological disorder due to COVID-19 of 53.3% and a relative precision of 8%. The calculated sample size was 150, and considering a 10% non-responsive rate, the required number of samples was 165 households, and the total number of samples collected was 291. We collected the data using a convenient sampling technique.

Data collection

The data collection was interview-based using a pre-designed, semi-structured questionnaire that contained questions on socio-demographic characteristics and used validated questionnaires like the Coronavirus Anxiety Scale (CAS), the Perceived Stress Scale (PSS), and the Generalized Anxiety Disorder (GAD) scale.

A self-reported mental health screening tool for dysfunctional anxiety linked to the coronavirus epidemic is called the CAS. The CAS's diagnostic capabilities (85% specificity and 90% sensitivity) are equivalent to those of analogous screening tools.

The most commonly used psychological tool for assessing stress perception is the PSS. It is a measure of how stressful a person perceives their life's circumstances to be.

As a screening device and severity predictor for GAD, Spitzer and colleagues (2006) designed the seven-item GAD scale. It was first developed to enhance awareness of GAD in primary care settings and is simple to score.

Data entry and analysis

We entered the collected data into Microsoft Excel (Microsoft Corporation, Redmond, WA), cleaned it, and analyzed it using SPSS version 21 (IBM Corp., Armonk, NY). Tables and figures depict the outcome variables as well as the presence of COVID-19-related anxiety and stress. We have taken socio-demographic characteristics as variables to describe characteristics of the study population, and to find associations with the outcome variables, we have used the chi-square test. We used Spearman's correlation to predict COVID-19 anxiety from the age factor.

## Results

The mean age of the study participants was 24.88 ± 7.94 years. More than half of the samples were from the female population (58.8%). More than half of the participants (185, 63.6%) were graduates or professionals, and 106 (36.4%) had education below a diploma or high school. The socio-economic status of the participants was as follows: 221 (75.9%) were in class 1, 47 (16.2%) were in class 2, and only 23 (7.9%) were in class 3. There were 207 (71.1%) urban participants and 84 (28.1%) rural participants.

More than half of the families (197, 67.7%) had no children and 46% were elderly. Among the samples, 34% had a previous history of COVID-19 infection, and 170 (58.4%) of the families had at least one chronic comorbidity among the family members (Table 1).

**Table 1:** Distribution of socio-demographic characteristics of the study population (n = 291) * Mean ± standard deviation.

Demographic characteristics	Frequency (%)
Age in years	24.88 ± 7.94*
Gender	
Male	120 (41.2)
Female	171 (58.8)
Education	
Diploma or high school	106 (36.4)
Graduate and above	185 (63.6)
Socioeconomic status	
Class 1	221 (75.9)
Class 2	47 (16.2)
Class 3	23 (7.9)
Residence	
Urban	207 (71.1)
Rural	84 (28.9)
Marital status	
Married	49 (16.8)
Unmarried	242 (83.2)
Presence of comorbidities in the family	
Yes	170 (58.4)
No	121 (41.6)
Presence of a child in the family	
Yes	94 (32.3)
No	197 (67.7)
Presence of the elderly in the family	
Yes	134 (46.0)
No	157 (54.0)
Previous history of COVID-19 in the family	
Yes	99 (34.0)
No	192 (66.0)

We described the distribution of anxiety and stress scale scores among the study population in Table 2. As per the CAS, 11% of the study samples were anxious. More than one-third of the study samples (30.9%) were severely anxious according to the GAD scale score. The PSS score implied that only 5.8% of the study participants were under high stress.

**Table 2:** Distribution of anxiety and stress scale scores among the study population (n = 291)

Scales	Frequency (%)
Coronavirus Anxiety Scale	
Not anxious	259 (89)
Anxious	32 (11)
Generalized Anxiety Disorder	
Mild anxiety	93 (32)
Moderate anxiety	96 (33)
Moderately severe anxiety	90 (30.9)
Severe anxiety	12 (4.1)
Perceived Stress Scale	
Low stress	27 (9.3)
Moderate stress	247 (84.9)
High stress	17 (5.8)

The CAS score was significantly associated with the residence (p = 0.049), marital status (p = 0.001), and previous history of COVID-19 (p = 0.016) of the study participants. Among the participants, the coronavirus anxiety stress was high among the rural population of 14 (16.7%) compared with the urban population of 18 (8.7%). The anxiety was acute among persons who were married (13, 26.5%) compared with persons who were unmarried (19, 7.9%). Persons with a history of COVID-19 infection expressed more anxiety (17.2%) compared with those without a past COVID-19 infection (7.8%) (Table 3).

**Table 3:** Association of Coronavirus Anxiety Scale scores with socio-demographic characteristics of the study population (n = 291)

Demographic characteristics	Not anxious	Anxious	P-value
Gender			
Female	154 (90.1)	17 (9.9)	0.492
Male	105 (87.5)	15 (12.5)	
Education			
Up to diploma or high school	90 (84.9)	16 (15.1)	0.091
Graduate or professionals	169 (91.4)	16 (8.6)	
Socioeconomic status			
Class 1	198 (89.6)	23 (10.4)	0.593
Class 2	42 (89.4)	5 (10.6)	
Class 3	19 (82.6)	4 (17.4)	
Residence			
Urban	189 (91.3)	18 (8.7)	0.049
Rural	70 (83.3)	14 (16.7)	
Marital status			
Married	36 (73.5)	13 (26.5)	<0.001
Unmarried	223 (92.1)	19 (7.9)	
Presence of comorbidities in the family			
Yes	155 (91.2)	15 (8.8)	0.160
No	104 (86)	17 (14)	
Presence of a child in the family			
Yes	80 (85.1)	14 (14.9)	0.142
No	179 (90.9)	18 (9.1)	
Presence of the elderly in the family			
Yes	117 (87.3)	17 (12.7)	0.395
No	142 (90.4)	15 (9.6)	
History of COVID-19 infection in the family			
Yes	82 (82.8)	17 (17.2)	0.016
No	177 (92.2)	15 (7.8)	

The PSS score was significantly associated only with the gender (p = 0.022) of the study participants. Among the participants, the perceived stress was high among females (7.6%) compared with males (3.3%) (Table 4).

**Table 4:** Association of Perceived Stress Scale scores with socio-demographic characteristics of the study population (n = 291)

Demographic characteristics	Low stress	Moderate stress	High stress	P-value
Gender				
Female	10 (5.8)	148 (86.5)	13 (7.6)	0.022
Male	17 (14.2)	99 (82.5)	4 (3.3)	
Education				
Up to diploma or high school	9 (8.5)	90 (84.9)	7 (6.6)	0.871
Graduate or professionals	18 (9.7)	157 (84.9)	10 (5.4)	
Socioeconomic status				
Class 1	19 (8.6)	190 (86)	12 (5.4)	0.889
Class 2	5 (10.6)	39 (83)	3 (6.4)	
Class 3	3 (13)	18 (78.3)	2 (8.7)	
Residence				
Urban	23 (11.1)	171 (82.6)	13 (6.3)	0.195
Rural	4 (4.8)	76 (90.5)	4 (4.8)	
Marital status				
Married	3 (6.1)	45 (91.8)	1 (2)	0.297
Unmarried	24 (9.9)	202 (83.5)	16 (6.6)	
Presence of comorbidities in family				
Yes	14 (8.2)	146 (85.9)	7 (5.8)	0.768
No	13 (10.7)	101 (83.5)	10 (5.9)	
Presence of a child in the family				
Yes	8 (8.5)	82 (87.2)	4 (4.3)	0.678
No	19 (9.6)	165 (83.8)	13 (6.6)	
Presence of the elderly in the family				
Yes	11 (8.2)	114 (85.1)	9 (6.7)	0.729
No	16 (10.2)	133 (84.7)	8 (5.1)	
History of COVID-19 infection in the family				
Yes	5 (5.1)	85 (85.9)	9 (9.1)	0.060
No	22 (11.5)	162 (84.4)	8 (4.2)	

The GAD scale score was also significantly associated with only the gender (p = 0.010) of the study participants. Among the participants, the GAD score was severe among the female population (4.7%) compared with the male population (3.3%) (Table 5).

**Table 5:** Association of Generalized Anxiety Disorder scale scores with socio-demographic characteristics of the study population (n = 291)

Demographic characteristics	Mild	Moderate	Moderately severe	Severe	P-value
Gender					
Female	51 (29.8)	47 (27.5)	65 (38)	8 (4.7)	0.01
Male	42 (35)	49 (40.8)	25 (20.8)	4 (3.3)	
Education					
Up to diploma or high school	34 (32.1)	39 (36.8)	29 (27.4)	4 (3.8)	0.69
Graduate or professionals	59 (31.9)	57 (30.8)	61 (33)	8 (4.3)	
Socioeconomic status					
Class 1	70 (31.7)	70 (31.7)	74 (33.5)	7 (3.2)	0.235
Class 2	17 (36.2)	18 (38.3)	10 (21.3)	2 (4.3)	
Class 3	6 (26.1)	8 (34.8)	6 (26.1)	3 (13)	
Residence					
Urban	64 (30.9)	68 (32.9)	65 (31.4)	10 (4.8)	0.76
Rural	29 (34.5)	28 (33.3)	25 (29.8)	2 (2.4)	
Marital status					
Married	10 (20.4)	20 (40.8)	19 (38.8)	0 (0)	0.067
Unmarried	83 (34.3)	76 (31.4)	71 (29.3)	12 (5)	
Presence of comorbidities in family					
Yes	46 (27.1)	61 (35.9)	58 (34.1)	5 (2.9)	0.077
No	47 (38.8)	35 (28.9)	32 (26.4)	7 (5.8)	
Presence of a child in the family					
Yes	32 (34)	28 (29.8)	29 (30.9)	5 (5.3)	0.777
No	61 (31)	68 (34.5)	61 (31)	7 (3.6)	
Presence of the elderly in the family					
Yes	43 (32.1)	46 (34.3)	38 (28.4)	7 (5.2)	0.707
No	50 (31.8)	50 (31.8)	52 (33.1)	5 (3.2)	
History of COVID-19 infection in the family					
Yes	25 (25.3)	30 (30.3)	37 (37.4)	7 (7.1)	0.055
No	68 (35.4)	66 (34.4)	53 (27.6)	5 (2.6)	

The age of the participants was significantly associated with all three stress scale scores, namely, GAD scale (r = -0.120, p = 0.040), CAS (r = 0.133, p = 0.024), and PSS (r = -0.209, p < 0.001) (Table 6).

**Table 6:** Correlation of age with GAD, PSS, and CAS scale scores (n = 291) GAD: Generalized Anxiety Disorder scale; PSS: Perceived Stress Scale; CAS: Coronavirus Anxiety Scale.

Scale	Correlation coefficient, r	P-value
Generalized Anxiety Disorder scale	-0.120	0.040
Perceived Stress Scale	-0.209	<0.001
Coronavirus Anxiety Scale	0.133	0.024

## Discussion

We carried out a cross-sectional study among the general population of the Perambalur district. We collected a total of 291 samples and estimated COVID-19 anxiety from July 2021 to October 2021. In the present study, most participants belonged to the same age group (mean age of 24 years), and most participants were females. Three-fourths of the participants belonged to socioeconomic class 1, and most of them were graduates. The primary aim of this study was to identify anxiety because of COVID-19 and determine the COVID-19 stressors.

In the present study, anxiety because of COVID-19 based on the CAS was 11%, and based on the GAD scale, it was 30.9% for moderate severity and 4.1% for severe anxiety. According to a study conducted in 2020 in China by Cao et al., the anxiety caused by COVID-19 was 24.9% [14]. In another study conducted by Wang et al. among general participants in 2020, the prevalence of psychological impact was 53.8% [15]. This study's findings contrast with our study's findings, where anxiety is much less prevalent. The probable reason for this difference may be because of the difference in the geographical location of the study.

In the current study, COVID-19 anxiety was found to be significantly related to the study participants' age, residence (p = 0.049), marital status (p = 0.001), and history of COVID-19 infection (p = 0.016). The generalized anxiety was also significantly associated with factors like gender (p = 0.010). Similarly, in a study conducted by Alzahrani et al. in Saudi Arabia, the anxiety caused by COVID-19 was found to be significantly associated with gender [16].

In another study conducted by Kantor et al., gender and residence were significantly associated with COVID-19 anxiety, and marital status was not associated [17]. This contrasts with our study findings, where age, gender, marital status, and residence were all significantly associated.

In the present study, perceived stress was significantly associated only with gender and age. But according to a study conducted by Sulistyawati et al., stress had a significant relationship with age, marital status, occupation, and income [18].

The following study has certain advantages. In the present study, we collected the data using a validated, semi-structured, standard tool that collected data on COVID-19 anxiety and stress. We counseled the participants who were anxious on ways to overcome anxiety and educated them about taking vaccines. Few studies have investigated COVID-19 anxiety and stress, which adds to the study's novelty. Since it was a community-based, cross-sectional study, the study findings have good validity and generalizability.

Limitations

The study is not without limitations. First, we gathered the data using a convenient sampling technique. Second, the study involved participants only from a single geographical area. No sub-group analysis was done pertaining to baseline characters. Although anxiety and stress were assessed using standardized scales, the evaluation was subjective in nature.

## Conclusions

Among the study samples, nearly one-third had a history of COVID-19 infection. The CAS score was significantly related to residence, marital status, and COVID-19 infection history. The study found that gender was the only factor associated with both the PSS score (p = 0.022) and the GAD scale score (p = 0.010) of the study participants. Many mental health conditions can be effectively treated at relatively low cost, yet the gap between people needing care and those with access to care remains substantial. Implementing government policies and programs to conduct a routine survey to detect anxiety and stress can provide a means of effective prevention.
